# A rare case of acute respiratory distress syndrome caused by use of gadolinium‐based magnetic resonance imaging contrast media

**DOI:** 10.1002/rcr2.483

**Published:** 2019-09-05

**Authors:** Yi Lee, Tzu Yi Chung, Hsu‐Chung Liu

**Affiliations:** ^1^ Division of Chest Medicine, Department of Internal Medicine Cheng Ching Hospital Taichung Taiwan, Republic of China; ^2^ Department of General Surgery MacKay Memorial Hospital Taipei Taiwan, Republic of China

**Keywords:** Acute respiratory distress syndrome, gadobutrol, gadolinium, magnetic resonance imaging contrast agent

## Abstract

Gadolinium‐based magnetic resonance imaging (MRI) contrast is generally considered to be stable and safe. Adverse reactions due to MRI contrast agents are classified into allergic‐like reactions and physiological reactions. Acute respiratory distress syndrome (ARDS) caused by gadolinium‐based MRI contrast is extremely rare. Due to the immediate and severe nature of ARDS, medical practitioners may seek after other aetiologies other than MRI‐contrast‐induced ARDS for patients' clinical manifestations such as acute‐onset difficulty of breathing. It is crucial to keep in mind the possibility of ARDS after gadolinium injection, as missing the diagnosis leads to a high mortality. A clear clinical scenario of ARDS induced by gadobutrol (Gadovist, Bayer Inc., Toronto, Canada) was presented in our patient who did not develop symptoms of anaphylaxis. We successfully managed the patient with methylprednisolone and bilevel positive airway pressure ventilation and the patient was discharged in stable condition on day 6.

## Introduction

Since 1988, gadopentetate dimeglumine debuted, GBCAs have been widely used and account for 30% of all magnetic resonance imaging (MRI) procedures up to date 1. Gadolinium is composed of paramagnetic compounds that possesses a high magnetic component and is most stable with unpaired electron. Unlike iodinated contrast media, MRI contrast agents such as gadolinium have few side effects, and rarely cause anaphylactoid reactions 2. To our best knowledge, only two cases of non‐cardiogenic pulmonary oedema induced by gadolinium‐based MRI contrast media have been reported 3, 4. Here, we present a Taiwanese woman who developed acute respiratory distress syndrome (ARDS) without anaphylactic symptoms after the use of gadobutrol and had a successful treatment with steroids and bilevel positive airway pressure (BiPAP) ventilation.

## Case Report

**Figure 1:**
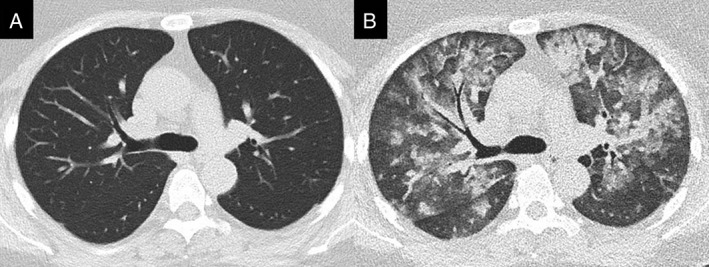
The chest computed tomography (CT) findings: (A) low‐dose computed tomography of health check‐up showed normal attenuation of bilateral lung parenchyma; (B) repeated CT scan 4 h later revealed multiple ground glass attenuation and airspace consolidation in bilateral lungs.

A 49‐year‐old woman (89.9 kg, 167.1 cm, body mass index: 32.3 kg/m^2^) without any past medical history, including heart failure, asthma, allergies, or immediate hypersensitivity reaction to any type of iodinated radiocontrast material, visited our hospital for a self‐paid medical imaging health check‐up‐package which includes the MRI‐upper abdomen imaging and low‐dose computed tomography (LDCT) of chest. Her initial non‐contrast LDCT of chest showed unremarkable finding (Fig. 1A). Two hours after LDCT, she underwent abdominal MRI after an injection of 15 mL (0.1 mL/kg body weight) of gadobutrol (Gadovist, Bayer Inc., Toronto, Canada). Ninety minutes after the injection of gadobutrol, she was found to have dyspnoea and cyanosis. Her vital signs were as follows: blood pressure 127/77 mmHg, pulse rate 100 bpm, respiratory rate 35/min, and oxygen saturation 60% by pulse oximetry.

**Figure 2:**
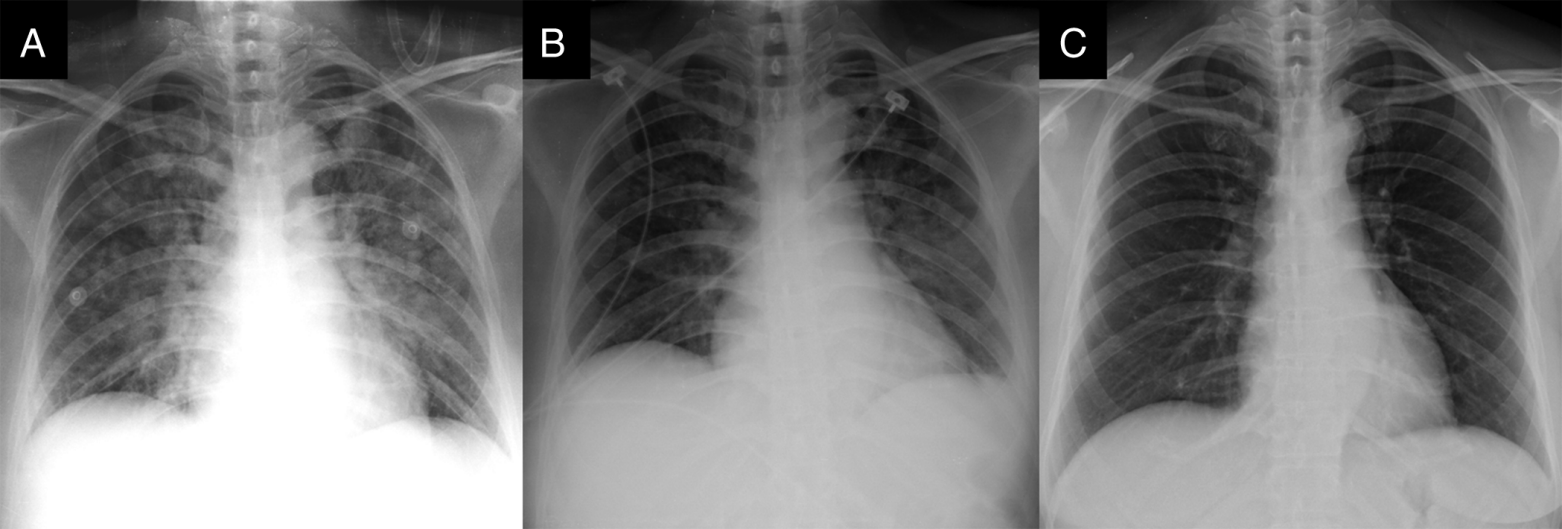
The serial chest radiographs: (A) breathlessness onset on day 1; (B) under bilevel positive airway pressure ventilation on day 3; (C) discharge on day 6.

At emergency room, physical examinations showed diffuse wheezes and use of accessory muscles of respiration. The chest radiograph (Fig. 2A) showed bilateral alveolar infiltrates and hilar haze suggestive of acute pulmonary oedema. Laboratory tests showed a serum creatinine of 0.4 mg/dL, a D‐dimer of 899 ng/mL, and a Brain Natriuretic Peptide (BNP) of 35.4 pg/mL. The repeated chest computed tomography scan showed multiple ground glass attenuation and consolidation in bilateral lungs (Fig. 1B). An echocardiography revealed no impaired left ventricular function or valvular defect. The initial arterial blood gas analysis showed a pH of 7.45, a partial pressure of carbon dioxide of 28.7 mmHg, and a partial pressure of oxygen (PaO_2_) of 48.6 mmHg, which was remarkable for severe oxygenation impairment with a PaO_2_/FiO_2_ ratio of 121.5 (FiO_2_: 40%). Under a diagnosis of MRI contrast‐induced ARDS, she was transferred into intensive care unit (ICU) where BiPAP ventilation with a 15/5 cm H_2_O pressure support was administered. Her hypoxaemia improved to a PaO_2_ level of 85 mmHg after the use of BiPAP ventilation. In addition, she received intravenous dexamethasone 5 mg immediately at emergency room and then switched to methylprednisolone injection with a maintenance dose of 1.5 mg/kg daily. During the ICU course, the patient got improvement from respiratory distress and hypoxaemia. The repeated chest radiograph on day 3 (Fig. 2B) revealed rapid resolution of airspace infiltrates in bilateral lungs. The patient was weaned successfully from BiPAP ventilation on day 4 and she was discharged with resolution of pulmonary infiltrates (Fig. 2C) on day 6.

## Discussion

Over the past two decades, since GBCA debuted, its use has been significantly increased 1. GBCA are considered to be stable and well tolerated by patients in clinical use. Many studies have postulated transmetallation hypothesis that free gadolinium is an inhibitor of some metabolic enzymes and the release of which leads to tissue damage 5. Awareness of GBCAs toxicity such as nephrogenic systemic fibrosis in chronic kidney disease patients has been raised. Recent studies strongly suggested gadolinium accumulation in tissue even in those with normal kidney function. However, adverse effects such as allergic reactions and non‐allergic reactions due to GBCAs based contrast medium are rarely reported. Regarding GBCA‐induced hypersensitivity reactions, Galera et al. reported that GBCAs of hyperosmolarity in nature may be IgE‐mediated rather than non‐specific histamine release comparing to iodinated contrast media 6. A recent meta‐analysis reported immediate allergic reactions in nine studies with a total of 716,978 administrations of GBCA, the overall rates of GBCA allergic‐like adverse events were 9.2 per 10,000 administrations 2.

ARDS is a sequence of an alveolar injury producing diffuse alveolar damage causing release of pro‐inflammatory cytokines, which damage the capillary endothelium and alveolar epithelium. With a variety of aetiologies and its acute course of lung injury, successfully identifying and managing ARDS is critical to reduce the high mortality rate 7. To our best knowledge, only two cases of gadolinium‐induced ARDS were reported 3, 4. The two previous cases were female patients without comorbidities or allergy history 3, 4, which is consistent with our patient. Park et al. 3 reported a patient who developed anaphylactic shock, angioedema of the lips, and pulmonary oedema 50 min after the injection of gadobutrol. Another case was a patient who also developed swelling of lips and uvula and pulmonary oedema 30 min after MRI‐contrast administration for a submandibular mass. Due to evident allergic reactions, both cases were immediately managed with intramuscular injection of epinephrine and intravenous dexamethasone under a diagnosis of severe allergic reaction or anaphylaxis. Herein, we present a patient who developed mild ARDS according to Berlin definition 8 and was successfully treated with BiPAP ventilation and intravenous methylprednisolone. Unlike the previous cases, our patient developed dyspnoea and central cyanosis at 90 min after gadolinium injection without signs of anaphylactic reactions such as skin rash, angioedema, or hypotension. Cardiogenic pulmonary oedema was excluded in this patient because of the normal results of BNP level and echocardiography. Therefore, we hypothesized that the cause of ARDS after the administration of gadobutrol in our case is more like idiosyncratic reaction. Following methylprednisolone administration and BiPAP ventilation, the patient recovered soon as previously described cases. For medical professionals in clinical practice, we believe that awareness should be raised for patients with immediate respiratory distress without any evident skin reactions or angioedema after the administration of gadolinium‐base contrast media.

According to a recent retrospective study by McDonald et al., several risk factors are identified for adverse reactions from gadolinium‐based contrast agent including women, 21–50 years of age, outpatient settings, abdomen and/or pelvis MRI imaging, and MRI contrast gadobutrol or gadobenate dimeglumine 9. Murphy et al. and Hunt et al. reported that patients who have a prior history of adverse reactions to iodinated contrast media have a higher frequency of occurrence of adverse reactions to gadolinium contrast 10, 11; however, this has not been addressed as a predictive variable in the model proposed by McDonald et al. 9. In addition, the mechanism under the interaction between iodinated contrast media and gadolinium‐based contrast is not well‐established. The reason may be the rarity of the adverse reactions in patients who had undergone both imaging examinations. Nevertheless, we strongly recommend that patients with allergic‐like or physiological reactions from gadolinium‐based contrast should avoid both gadolinium‐based contrast media and iodinated contrast media. Primary prevention such as giving patients alert card or skin testing are also suggested 12.

In conclusion, severe complications related to gadolinium‐based contrast are sparse in healthy population without renal impairment for its high‐safety profile. However, it is crucial to document severe allergic reaction or idiosyncratic reaction such as ARDS and provide these patients with appropriate treatment and prevention methods.

### Disclosure Statement

Appropriate written informed consent was obtained for publication of this case report and accompanying images.
